# Primary Clitoral Melanoma: Personalized Therapeutic Strategies Informed by Clinical Evidence and Systematic Review

**DOI:** 10.3390/jpm16020070

**Published:** 2026-01-31

**Authors:** Anna Pitsillidi, Laura Vona, Guglielmo Stabile, Günter Noé

**Affiliations:** 1 Department of Obstetrics and Gynaecology, Rheinland Klinikum Neuss, Preußenstrasse 84, 41464 Neuss, Germanyguenter.noe@uni-wh.de (G.N.); 2 Department of Obstetrics and Gynaecology, University of Witten Herdecke, 58448 Witten, Germany; 3 Department of Medical and Surgical Sciences, Institute of Obstetrics and Gynaecology, University of Foggia, 71122 Foggia, Italy; guglielmost@gmail.com

**Keywords:** clitoral melanoma, clitoris melanoma, primary clitoral melanoma, vulvar melanoma, clitoris

## Abstract

**Introduction:** Mucosal melanomas are rare, and vulvar melanoma is typically diagnosed at an advanced stage with aggressive behavior and poor prognosis. The clitoral region adds challenges due to its functional importance and lack of a dedicated staging system, requiring individualized management. This review evaluates current evidence on prognosis with emphasis on clitoral involvement and highlights diagnostic and therapeutic challenges, underscoring the need for personalized strategies and prospective multicentre studies. **Materials and Methods:** A systematic review, registered in PROSPERO (CRD420251151187), was conducted per PRISMA guidelines across PubMed, Scopus, Embase, and Web of Science, including English-language case reports and series of primary clitoral melanoma published until August 2025, with no historical limits. **Results:** 15 cases from 10 studies were identified. The mean patient age was 60 years, with most tumors presenting at advanced stages (median Breslow thickness of 8 mm, frequent ulceration). Immunohistochemical markers and gene mutations are rarely investigated in reported cases. All patients underwent surgery, with variable lymph node assessment; adjuvant therapy was rarely used. Recurrence occurred in nearly one-third of cases, sometimes more than 10 years after initial treatment. **Conclusions:** Primary clitoral melanoma is extremely rare and often diagnosed late, underscoring the need for heightened clinical awareness. Wide local excision with organ preservation is preferred, and bilateral sentinel lymph-node biopsy can improve staging. The absence of a dedicated staging system and limited systemic evidence highlight the need for standardized protocols. Emerging molecular and immunologic approaches are promising, but prospective multicentre studies are essential to guide management.

## 1. Introduction

Mucosal melanomas are uncommon, accounting for roughly 3% of all melanomas, in contrast to the far more frequent cutaneous forms [1]. Among these, vulvar melanoma is particularly uncommon and highly aggressive, accounting for only 5–10% of vulvar malignancies, yet it remains the second most common malignant tumour of the vulva after squamous cell carcinoma [2,3]. Although it shares some histopathological and molecular features with cutaneous melanoma, it displays a distinct clinical behaviour, with poorer prognosis and variable treatment response [4]. The median age at diagnosis exceeds 50 years, and in most cases, the disease is detected at an advanced stage, with significant negative impact on overall survival [5].

Epidemiologically, vulvar melanoma represents an extremely rare malignancy, accounting for approximately 0.1–0.2 per 100,000 women annually, with incidence rates that have remained relatively stable over recent decades [5,6]. Compared with cutaneous melanoma, vulvar melanoma shows significantly higher disease-specific mortality, with five-year survival rates ranging from 27% to 60% depending on stage at diagnosis [5,7]. Its rarity and biological heterogeneity contribute to the scarcity of robust prognostic data and limit the development of standardized management approaches.

Delayed diagnosis is frequent, reflecting the rarity of the condition, low clinical suspicion, and its often-nonspecific presentation. Early symptoms tend to be vague and may include vulvar discomfort, pruritus, dyspareunia, dysuria, abnormal bleeding, or the presence of a palpable lesion [6]. On examination, vulvar melanoma may present as a pigmented macule or papule with irregular borders, or as a nodular lesion with variegated colour—appearances that readily mimic benign vulvar conditions and contribute to delayed diagnosis [8]. Consequently, tumours are often larger, with greater Breslow thickness and regional lymphatic involvement at presentation, both of which contribute to a worse prognosis [5,7].

Vulvar melanoma most frequently arises in the labia majora, followed by the clitoral hood and labia minora, but it can also present as a multifocal lesion [9]. Therefore, surgery in this site is especially challenging, given the functional, sexual, and aesthetic relevance of the vulvar region. Achieving adequate oncological margins must be carefully balanced against preserving anatomical function and quality of life [10]. A further challenge is the absence of a dedicated staging system for clitoral or vulvar melanoma. Current practice applies cutaneous melanoma criteria, which may underestimate the aggressive biology of mucosal sites [9,10].

Available evidence on prognostic factors and treatment strategies is limited and based primarily on case reports and case series. Reported prognostic indicators include Breslow thickness, presence of ulceration, lymph node involvement and perineural invasion [11]. The rarity of these tumours and heterogeneity of available studies continue to limit development of reliable prognostic models and evidence-based treatment algorithms. Advances in molecular oncology allow treatment tailoring based on tumour genetics and immune markers. Yet, for rare mucosal sites like the clitoris, personalized management strategies remain poorly defined.

This review aims to critically evaluate current evidence on the prognosis of vulvar melanoma, with particular emphasis on clitoral involvement and the associated diagnostic, surgical, and therapeutic challenges. By identifying existing gaps in knowledge and clinical practice, we highlight the need for personalized management strategies and prospective multicentre studies focused on this rare malignancy.

## 2. Materials and Methods

### 2.1. Search Strategy

**Figure 1:**
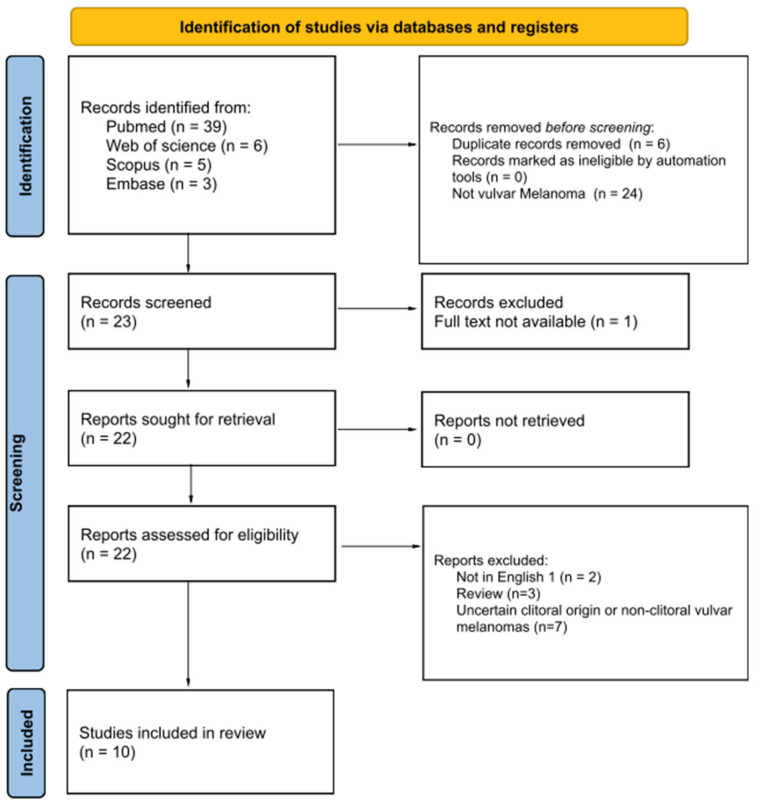
PRISMA flow diagram.

This systematic review was conducted in accordance with the PRISMA guidelines for systematic reviews (Supplementary Table S1) [12]. Two independent reviewers (L.V. and A.P.) performed a comprehensive literature search of the Web of Science, Scopus, Embase and MEDLINE (PubMed) databases, including all studies published up to August 2025, with no historical limits. The search strategy combined the following keywords and MeSH terms: “clitoral melanoma,” “clitoris melanoma,” “primary clitoral melanoma,” “vulvar melanoma AND clitoris” and “vulvar melanoma”. The study selection process is detailed in the PRISMA flow diagram (Figure 1).

### 2.2. Eligibility Criteria

Eligible study designs included case reports, randomized controlled trials, prospective controlled studies, prospective cohort studies, retrospective studies, and case series. Only full-text articles published in English were included. However, the available literature consisted solely of case reports and case series. Articles not published in English, systematic reviews, meta-analysis, letters to the editor, and conference abstracts were excluded. However, reference lists of relevant reviews were manually screened to identify additional eligible studies. Importantly, only cases of primary clitoral melanoma with a clearly identifiable origin in the clitoris were included. Vulvar tumours involving the clitoris but without a clearly documented clitoral origin were excluded.

### 2.3. Data Extraction and Risk-of-Bias Assessment

All records identified through database searches were screened for publication year, citation details, title, authorship, abstract, and full text. Duplicate records were manually identified and removed independently by two reviewers (A.P. and L.V.). Titles and abstracts of the remaining articles were independently screened by the same reviewers to exclude irrelevant studies. Full texts of potentially eligible studies were then independently assessed for inclusion. Discrepancies were resolved by discussion and consensus. The methodological quality of included studies was assessed using the Joanna Briggs Institute (JBI) Critical Appraisal Checklist for Case Reports (Supplementary Table S2). This study has been registered in the PROSPERO database (registration number: CRD420251151187). The inclusion of only case reports and case series in this review presents a risk of bias.

### 2.4. Data Synthesis and Statistical Analysis

Data extracted included patient demographics, tumour characteristics, treatment, recurrence and outcomes. Where possible, continuous variables were reported as means, while discrete and dichotomous variables were presented as percentages. Due to the low number of patients in our review, some data are presented descriptively.

## 3. Results

A total of 53 records were identified through the database search (39 from PubMed, 6 from Web of Science, 5 from Scopus, and 3 from Embase). After removal of duplicates and exclusion based on eligibility criteria, 10 publications describing 15 individual cases of primary clitoral melanoma were included in the final review (Table 1).

### 3.1. Patient Characteristics

Patient age ranged from 18 to 82 years (mean 57 years); six patients were ≤50 years old. Most tumours were diagnosed at an advanced stage, commonly pT4b (stage IIC). Li et al. reported a very young patient (18 years old) who presented with stage IV disease. Earlier reports used FIGO or previous versions of the AJCC/UICC staging systems. Breslow thickness was reported in nine cases and ranged from 5 mm to 28 mm (median 8 mm). Ulceration was frequent. Immunohistochemical positivity was reported in three studies [10,13,16] and pathogenic mutations in two [15,16], with positivity for SOX10, S100, HMB45, MART-1, and Melan-A, and mutations involving PTEN, NRAS, and PD-1.

### 3.2. Treatment Approaches

All patients underwent surgery. Procedures included wide local excision with sentinel lymph-node biopsy [13,16,17,18], partial or radical vulvectomy with or without lymphadenectomy [14,19,21,22], and partial vulvectomy with sentinel node biopsy [10]. A shave biopsy was performed for the patient described by Li et al., as metastatic disease had been detected [15]. Adjuvant therapy was administered in 4 of 15 cases (27%), consisting of experimental regimens in historical series, interferon [18], immune checkpoint blockade with pembrolizumab [16] or dacarbazine, a chemotherapeutic agent [15].

### 3.3. Recurrence and Outcomes

**Figure 2:**
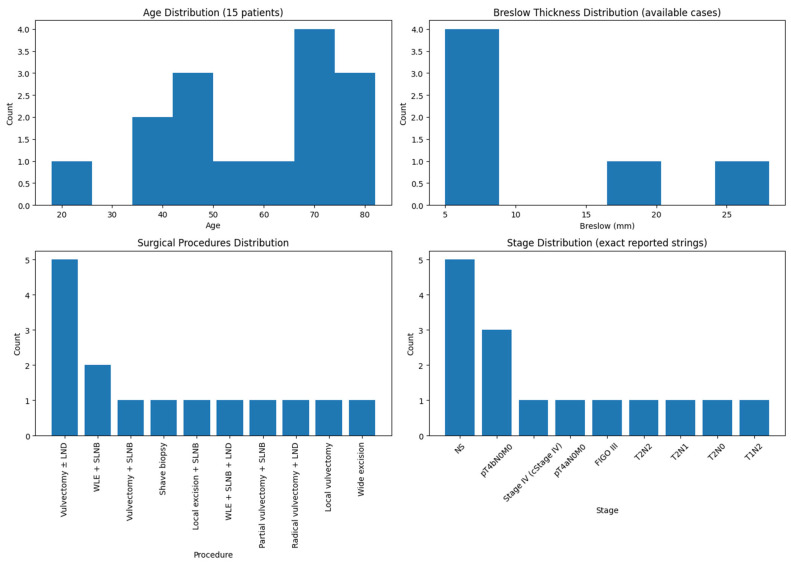
Visual Overview of Age, Breslow Thickness, Surgical Approaches, and Staging.

Disease recurrence occurred in 5 of 15 patients (33%), with time to relapse ranging from 9 months to 12 years. Recurrences involved distant organs (lung and liver) as well as locoregional areas such as vulva, vagina, mons pubis, and inguinal lymph nodes. No recurrences were reported in patients with follow-up shorter than 12 months or in those monitored for more than 12 years (e.g., White et al. [17]; Cascinelli et al., one patient alive at 9 years [20]). Reported follow-up across the cohort ranged from 1 month to 18 years. Long-term survivors (>5 years) were documented by Piura et al. (12 years) [19], Cascinelli et al. (up to 9 years) [20], Iwasaki et al. (9 years) [13], and only one patient by Janovski et al. (18 years) [21], whereas early mortality (<3 years) was common in older series. In the case reported by Li et al., the patient presented with multiple abdominal lesions, as well as involvement of both lungs, kidneys, bones, and numerous lymph nodes and she died after 41 days due to multi-system organ failure and, ultimately, cardiac arrest [15]. Figure 2 provides a visual overview of patient age, Breslow thickness, surgical approaches, and disease staging.

## 4. Discussion

The findings of this systematic review demonstrate that primary clitoral melanoma is exceptionally rare, with only 15 well-documented cases reported over more than six decades. Across the published literature, tumours were typically diagnosed at advanced stages, with substantial Breslow thickness, frequent ulceration, and heterogeneous reporting of immunohistochemical and molecular markers. Treatment modalities varied widely, and recurrence occurred in approximately one-third of cases—sometimes more than ten years after initial therapy. These observations highlight the need for improved diagnostic awareness, individualized surgical planning, and prolonged follow-up. To contextualize these findings, we also present an illustrative case from our institution, which reflects many of the challenges observed in the published literature.

### Case Presentation

A 78-year-old Caucasian woman presented with a pigmented clitoral lesion that had progressively enlarged over the preceding month and intermittently bled. Her medical history included chronic gastritis, gastro-oesophageal reflux disease, and multinodular struma, and her surgical history comprised two caesarean deliveries and a total hysterectomy. There was no family history of melanoma or other malignancy. On pelvic examination, a solitary blue–black, exophytic mass measuring approximately 2 × 2.5 cm was observed on the clitoral glans, with irregular yet well-defined borders, and no palpable inguinal or pelvic lymphadenopathy.

**Figure 3:**
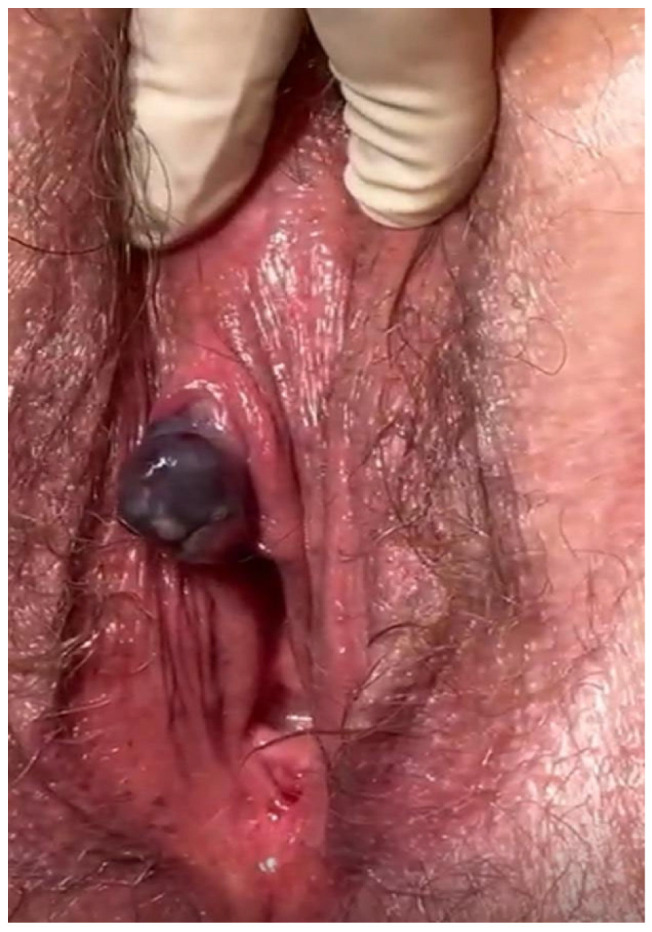
Solitary blue–black, exophytic mass.

An incisional biopsy confirmed malignant melanoma. Contrast-enhanced computed tomography (CT) of the abdomen and thorax showed no evidence of regional or distant metastases. The patient subsequently underwent wide local excision of the tumour, which revealed positive margins (R1). A re-excision achieved complete microscopic clearance (R0). Final histopathological examination demonstrated malignant melanoma with perineural invasion (Pn1), no evidence of lymphovascular invasion (V0), and Clark level IV. According to the AJCC 8th edition, the tumour was staged as pT4a pN0 cM0 (Figure 3). Molecular testing (including BRAF) was not performed, in accordance with institutional practice for early-stage mucosal melanoma and given the patient’s decision to decline additional interventions.

Despite multidisciplinary counselling regarding sentinel lymph-node biopsy and adjuvant systemic therapy, the patient declined further treatment. Given her age, comorbidities, and personal priorities, she opted for a conservative approach—highlighting the importance of shared, patient-centred decision-making in rare oncologic conditions. At six-month follow-up, clinical examination and cervical cytology were negative for local recurrence or metastatic disease.

Primary melanoma of the clitoris is very rare and represents a diagnostic and therapeutic challenge. Our case illustrates the typical clinical scenario of delayed presentation and advanced local disease. Although the duration of symptoms was brief, the lesion already demonstrated significant thickness and perineural invasion—findings consistent with prior reports of vulvar melanoma, where nonspecific symptoms and benign-appearing pigmented lesions often postpone diagnosis and contribute to worse outcomes [5,6,8].

**Figure 4:**
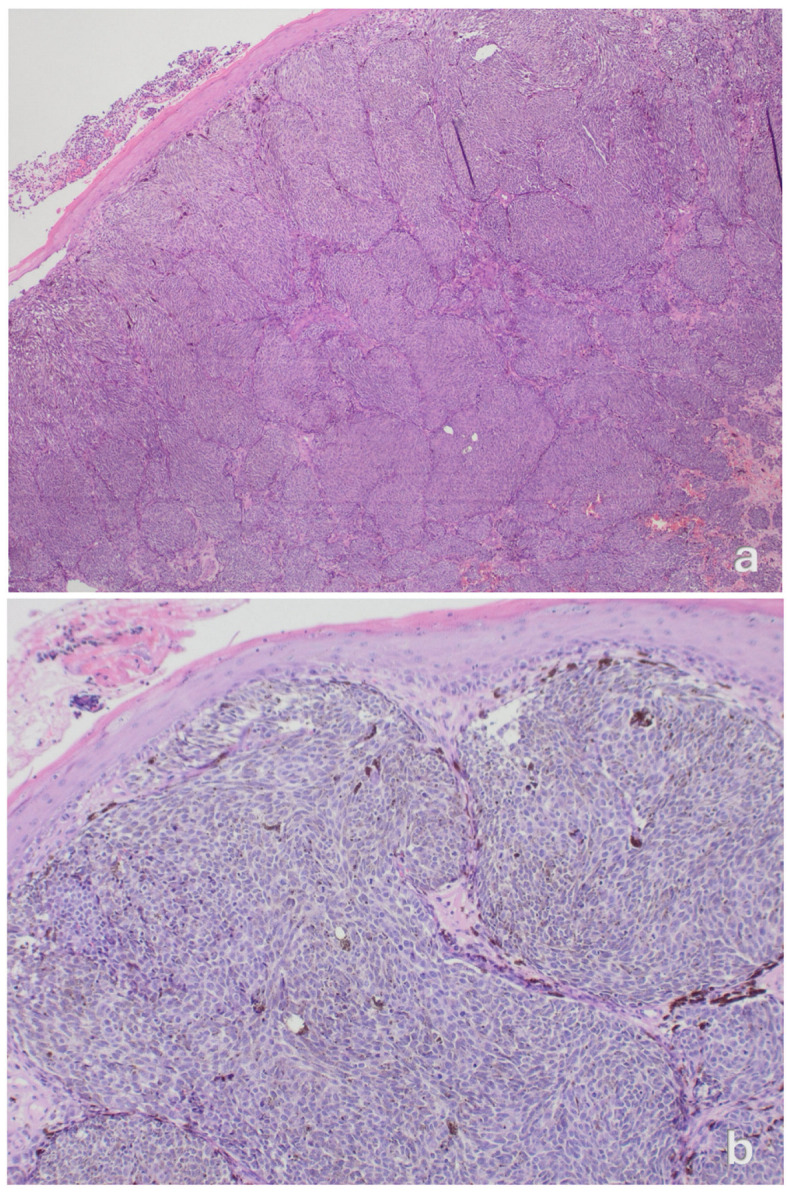

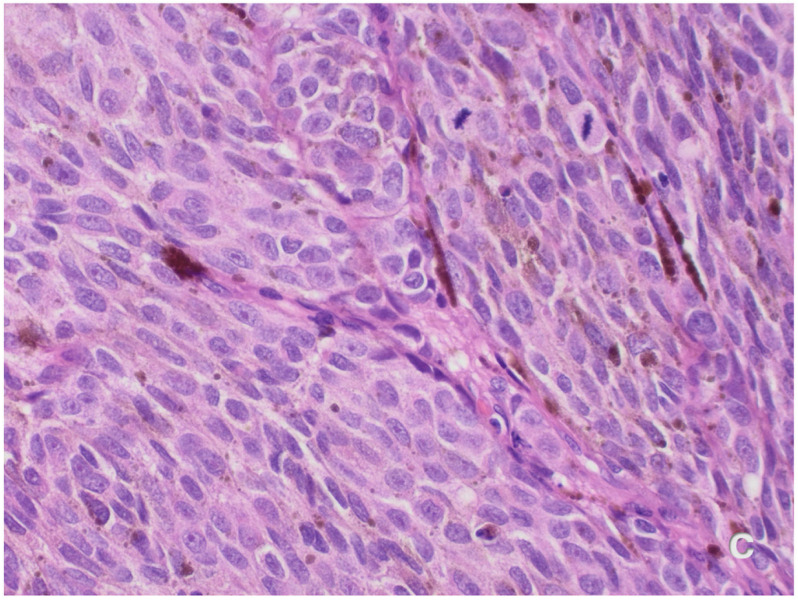
Primary clitoral melanoma. (**a**) Low-power H&E section (original magnification ×40) shows a nodular, sheet-like dermal proliferation of atypical melanocytes beneath the squamous epithelium. (**b**) Medium-power view (×100) highlights confluent nests of tumour cells with scattered melanin pigment. (**c**) High-power view (×400) demonstrates pleomorphic melanocytes with prominent nucleoli and intracytoplasmic melanin.

Our systematic review highlights the scarcity of available evidence: only 15 well-documented cases were identified over more than six decades. Patients were predominantly older (mean age 60 years), and most tumours were diagnosed at an advanced stage with substantial Breslow thickness (median 8 mm). Ulceration was frequently observed, and approximately one-third of patients developed disease relapse, sometimes after a prolonged interval following the initial therapy. These findings emphasize the necessity for extended surveillance, given that relapses have been recorded over ten years after initial therapy. Hematoxylin and eosin staining is shown in Figure 4.

Surgical management remains the cornerstone of therapy, although the optimal extent of excision and nodal evaluation is still debated. Our patient underwent wide local excision, in keeping with contemporary practice that favours organ preservation when negative margins can be achieved. SLNB was recommended but declined. Despite the relative lack of high-quality supporting evidence, SLNB is currently recommended, as it enables accurate assessment of regional nodal involvement with lower morbidity compared to complete lymphadenectomy; notably, a negative SLNB may allow clinicians to safely avoid more extensive nodal dissection [23]. The midline location of the clitoris results in bilateral lymphatic drainage, increasing the risk of bilateral nodal metastasis and complicating nodal staging [18]. As a result, surgical planning often requires bilateral SLNB or inguinal lymphadenectomy because of the potential for bilateral lymphatic drainage [22,24]. Achieving oncologic control while preserving sexual and urinary function is extremely critical in the clitoral region, highlighting the importance of multidisciplinary planning. When wider excisions are necessary, reconstructive techniques—including local advancement flaps and V-Y fasciocutaneous flaps—can help restore clitoral hood or anterior vulvar tissue while maintaining sexual and urinary function [25,26]. Precision oncology offers new opportunities for mucosal and clitoral melanomas. Mutation testing and PD-L1 assessment may guide systemic therapy, while immune-checkpoint inhibitors and individualized surgical decisions highlight the role of personalized care despite limited molecular evidence [4].

Historically, adjuvant treatment strategies have varied widely, from experimental protocols to interferon. More recently, reports have described the use of immune-checkpoint inhibitors such as pembrolizumab [16], mirroring advances in melanoma therapy overall. Although evidence remains limited to single cases, these agents may be valuable for advanced or recurrence disease and merit consideration for eligible patients or within clinical trials.

A further challenge is the absence of a dedicated staging system for clitoral or vulvar melanoma. The clitoral region is an anatomical transition zone between keratinized cutaneous epithelium and non-keratinized mucosa. Although clitoral melanoma is not formally classified as a mucosal subtype, it is often approached as such because of its location, aggressive behaviour, and clinical resemblance to mucosal melanoma [11,27]. Nevertheless, staging and treatment generally follow cutaneous melanoma guidelines, reflecting the vulva’s partial origin from skin [9,10]. This highlights the unique challenges of staging and treatment at this site and underscores the need for management strategies tailored to its dual characteristics. Historically, as seen in Piura et al. case report, the absence of specific staging systems for vulvar melanoma led to the adoption of the FIGO classification (International Federation of Gynecology and Obstetrics), originally developed for vulvar squamous cell carcinoma [19]. The 7th edition of the UICC/AJCC TNM classification (Union for International Cancer Control, American Joint Committee on Cancer, Tumour–Node–Metastasis) (2010) introduced a distinct staging system for mucosal melanomas of the head and neck, but it does not extend to melanomas of the female genital tract, including vulva and vagina [28]. As a result, staging of vulvar melanomas continues to rely on systems originally developed for cutaneous melanoma [13,16,17,20,29], which may lack prognostic precision when applied to mucosal sites [27,30].

Given the absence of a dedicated staging system for vulvar and clitoral melanoma, our findings underscore an important clinical gap. Current reliance on cutaneous melanoma criteria may underestimate the biological aggressiveness of mucosal sites and fails to reflect the unique anatomical considerations of the clitoris, including bilateral lymphatic drainage and its functional relevance. The substantial proportion of patients presenting with advanced T stages and the occurrence of very late recurrences highlight the need for a staging framework that integrates mucosal behavior, local anatomical complexity, and long-term risk. Future research should focus on multicenter prospective registries to refine prognostic factors specific to clitoral melanoma and to develop staging criteria that more accurately stratify risk and guide surgical and systemic management.

The main limitation of this study is the extremely small number of well-documented cases available, resulting in heterogeneous reporting of staging, pathology, and treatment. Key data such as immunohistochemistry, genetics, and long-term follow-up were inconsistently described across studies, limiting comparability. A visual summary of key points and management strategies is presented in Table 2.

## 5. Conclusions

Primary clitoral melanoma is an exceptionally rare malignancy that presents diagnostic and therapeutic challenges. Our analysis underscores the need for heightened clinical vigilance, as delayed detection and advanced tumour thickness are common. Wide local excision with organ preservation remains the preferred approach, and bilateral sentinel lymph-node biopsy should be considered to improve staging accuracy.

The lack of a dedicated staging system and the limited evidence on systemic treatments highlight the necessity for standardized protocols. Molecular profiling and immune-checkpoint inhibitors show promise but require further validation. Prospective registries and multicentre studies are essential to refine prognostic models and develop evidence-based management strategies tailored to this unique anatomical site.

Our case also illustrates that, in selected patients of advanced age or with significant comorbidities, exclusive surgical management may represent an appropriate and personalized therapeutic option when aligned with patient preferences and multidisciplinary consensus.

## Figures and Tables

**Table 1:** Reported Cases of Primary Clitoral Melanoma.

Study (Year)	Age	Stage	Breslow (mm)	Immunohistochemical Positivity—Pathogenic Mutations	Initial Surgery	Adjuvant Therapy	Recurrence Site(s)	Recurrence Treatment	Follow-Up
Iwasaki et al. 2024 [13]	82	pT4bN0M0, pStage IIC	18, ulcerated	/	Wide local excision + sentinel node biopsy	None	4 yrs: right lung; 9 yrs: liver	Pneumectomy + Pembrolizumab; Hepatectomy + Pembrolizumab	9 years
Fuchs et al. 2021 [14]	69	NS	/	/	Simple Vulvectomy + radical vulvectomy and bilateral inguinal/femoral sentinel lymph node dissection	None	6 yrs: vulva 7 yrs: vulva 11 yrs: vulva 12 yrs: vulva + periurethral area 16 yrs: periurethral area	Partial vulvectomy + topical imiquimod Nivolumab	17 years
Li et al. 2021 [15]	18	cStage IV	/	SOX positivity—NRAS and PTEN mutation	Shave biopsy	Dacarbazine	/	/	Death after 41 days
Szlachta-McGinn et al. 2021 [16]	52	pT4bN0M0; pStage IIC	28, ulcerated	SOX10, S100, HMB45, MART1 positivity—PD-L1 mutation	Local excision (positive margins) → sentinel node biopsy and wide local excision after 6 weeks	Pembrolizumab	None reported	None	NS
White et al. 2019 [17]	67	pT4aN0M0; pStage IIB	8, not ulcerated	Melan-A and S-100 positivity	Wide local excision + sentinel node biopsy after 12 weeks	None	None reported	None	1 year 3 months
Takahashi et al. 2015 [18]	66	Not specified	7.5	/	Wide local excision + sentinel node biopsy + left inguinal lymphadenectomy	β-interferon	None reported	None	6 months
Kost’álová et al. 2007 [10]	77	pT4bN0M0; pStage IIC	5, ulcerated	/	Partial vulvectomy + sentinel node biopsy	None	None reported	None	9 months
Piura et al. 1999 [19]	46	FIGO Stage III	5	/	Radical vulvectomy + distal urethra resection + bilateral lymphadenectomy	None	6 yrs: vulvar; 10 yrs: vagina; 11 yrs: vagina; 12 yrs: widespread	Multiple local excisions + vaginal wall brachytherapy	12 years (death)
Cascinelli et al. 1970 [20]	1. 46 2. 71 3. 39 4. 74 5. 49	1.T2N2 2.T2N1 3.NS 4.T2N0 5.T1N2	NS	/	Cases 1, 2, 4, 5: Vulvectomy ± bilateral lymph node dissection; Case 3: Radiotherapy	Lipidol 1311	/	/	1: 1y5m (death); 2: 1y8m (death); 3: 9y (alive); 4: 4y (other cause); 5: 1y (alive)
Janovski et al. 1962 [21]	59 & 37	NS	NS	/	1: Local vulvectomy; 2: Wide excision	None	1: 1 yr mons pubis; 2y inguinal nodes| 2: 9 months inguinal node; 2 yrs inguinal node	1: Lymphadenectomy (refused further treatment); 2: Lymph node excision	1: 2y4m (death); 2: 18y

NS = not specified. Staging is reported as in the original articles. Follow-up is from initial diagnosis.

**Table 2:** Visual summary.

Category	Key Points/Strategies
Epidemiology & Presentation	Extremely rare; only 15 well-documented cases over >60 years. Typically older patients (mean age ~57). Often diagnosed late; thick and ulcerated tumors.
Pathology & Disease Characteristics	High Breslow thickness (median 8 mm), ulceration, perineural invasion. One-third relapse; recurrences > 10 years → long-term surveillance.
Surgical Management	Wide local excision; organ preservation when margins negative. Must balance oncologic control with sexual/urinary function.
Nodal Assessment	SLNB recommended; lower morbidity than full lymphadenectomy. Bilateral drainage → often bilateral SLNB or inguinal dissection.
Immunohistochemical positivity Pathogenic mutations	Immunohistochemical positivity reported in three studies. Common markers: SOX10, S100, HMB45, MART-1, Melan-A. Pathogenic mutations described in two reports, involving PTEN, NRAS, and PD-1. Supports diagnosis and may guide selection of systemic therapies in a precision-oncology approach.
Adjuvant & Systemic Therapy	Historically interferon/experimental. Immunotherapy (e.g., pembrolizumab) reported. Chemotherapeutic agent (e.g., dacarbazine) Precision oncology: mutation testing, PD-L1 assessment.
Staging Challenges	No dedicated staging system. Behaves like mucosal melanoma but staged as cutaneous. Current systems may lack prognostic accuracy.
Follow-up & Prognosis	High risk of late recurrence → extended follow-up required.
Research Needs	Prospective registries and multicentre studies needed.

## Data Availability

The original contributions presented in the study are included in the article. Further inquiries can be directed to the corresponding author.

## Supplementary Materials

The following supporting information can be downloaded at: https://www.mdpi.com/article/10.3390/jpm16020070/s1, Table S1: PRISMA_2020_checklist; Table S2: JBI Critical Appraisal Checklist for Case Reports.

## Abbreviations

SLNB	sentinel lymph-node biopsy
H&E	Hematoxylin and Eosin
Yrs	years
FIGO	International Federation of Gynecology and Obstetrics
UICC	Union for International Cancer Control, American Joint Committee on Cancer, Tumour–Node–Metastasis
AJCC	American Joint Committee on Cancer
TNM classification	Tumour–Node–Metastasis
