# Supplementary material for: Primary Clitoral Melanoma: Personalized Therapeutic Strategies Informed by Clinical Evidence and Systematic Review
Source: J Pers Med. 2026 Jan 31;16(2):70. doi: 10.3390/jpm16020070 (PMC12941946; doi:10.3390/jpm16020070)
Supplement: Supplementary file 1 [file jpm-16-00070-s001.zip › Table S2-JBI Critical Appraisal Checklist for Case Reports.pdf]

Author, Year	Title	D1	D2	D3	D4	D5	D6	D7	D8
Hitoshi Iwasaki, 2024	Robot-assisted laparoscopic hepatectomy for liver metastasis from clitoral malignant melanoma: a case report	YES	YES	YES	YES	YES	YES	YES	YES
Alec Szlachta-McGinn, 2021	Management of Clitoral Melanoma Presenting as an Exophytic Clitoral Mass: A Case Report and Review of the Literature	YES	YES	YES	YES	YES	YES	UNCLEAR	YES
Bradley White, 2019	Stage Two Malignant Melanoma of the Clitoris: A Case Report	YES	YES	YES	YES	YES	YES	YES	YES
Chiaki Takahashi, 2015	Malignant melanoma on female clitoris with bidirectional upper and lower lymphatic flow	YES	YES	YES	YES	YES	YES	YES	YES
Flavia Baterca, 2008	Amelanotic vulvar melanoma: case report and review of the literature	YES	YES	YES	YES	YES	YES	YES	YES
Marie Kosťálová, 2007	Melanoma clitoridis	YES	YES	YES	YES	YES	YES	YES	YES
Benjamin Piura, 1999	Malignant melanoma of the vulva: report of six cases and review of the literature	YES	YES	YES	YES	YES	YES	YES	YES
Natale Cascinelli, 1970	MALIGNANT MELANOMA OF THE VULVA	YES	UNCLEAR	YES	UNCLEAR	YES	YES	YES	YES
N.A. Janovski, 1962	Malignant melanoma of the vulva	YES	YES	UNCLEAR	UNCLEAR	YES	YES	YES	YES
